# Joint Association of Arterial Stiffness and Depression With New‐Onset Self‐Reported Chronic Obstructive Pulmonary Disease Among Elderly Chinese Population

**DOI:** 10.1002/brb3.71200

**Published:** 2026-01-19

**Authors:** Qinqin Shen, Xingyun Zhu, Yu‐Jun Xiong, Tianshui Li, Tian Lv, Hongxia Wang

**Affiliations:** ^1^ Department of Respiratory and Critical Care Medicine Beijing Jishuitan Hospital, Capital Medical University Xicheng District, Beijing People's Republic of China; ^2^ Department of Endocrinology Beijing Jishuitan Hospital, Capital Medical University Xicheng District, Beijing People's Republic of China; ^3^ Department of Gastroenterology, Beijing Hospital, National Center of Gerontology, Institute of Geriatric Medicine Chinese Academy of Medical Sciences Beijing P.R. China; ^4^ Department of Neurology Zhuji Affiliated Hospital of Wenzhou Medical University Zhuji China; ^5^ Department of Respiratory and Critical Care Medicine Zhuji Affiliated Hospital of Wenzhou Medical University Zhuji China

**Keywords:** CHARLS, COPD, depression, ePWV

## Abstract

**Background:**

Chronic Obstructive Pulmonary Diseases (COPD) impose a substantial global health burden, yet the joint impact of arterial stiffness and depression on their incidence remains underexplored.

**Methods:**

This cohort study analyzed adults aged ≥45 years from the China Health and Retirement Longitudinal Study (2011–2018). Participants with baseline COPD or missing data were excluded. Cox proportional hazards models assessed associations, while mediation analysis evaluated bidirectional roles of the estimated pulse wave velocity (ePWV) and 10‐item Center for Epidemiologic Studies Depression Scale (CESD‐10) in new‐onset COPD.

**Results:**

Over 7 years, 718 participants developed COPD. ePWV (HR = 1.11, *P* < 0.0001) and depression (HR = 1.63, *P* < 0.0001) independently increased risk, with the highest hazard in comorbid cases (HR = 2.17, *P* < 0.0001). ePWV mediated 1.7% of depression's effect, while depression mediated 4.8% of ePWV's impact (*P* < 0.05).

**Conclusion:**

Higher ePWV and depressive symptoms were independently associated with incident chronic obstructive pulmonary disease. The observed mediation effects were statistically significant but small in magnitude and should be interpreted as exploratory rather than clinically meaningful. These findings are hypothesis‐generating and warrant confirmation using objective measurements and causal study designs.

## Graphical abstract

Arterial stiffness and depressive symptoms independently and jointly increased the risk of new‐onset Chronic Obstructive Pulmonary Disease (COPD) in older Chinese adults. Their small bidirectional mediation effects suggest interconnected vascular and psychological pathways contributing to COPD development.

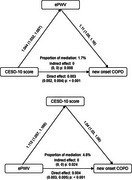

## 1 Introduction

Chronic Obstructive Pulmonary Disease (COPD) is a progressive respiratory disorder characterized by persistent airflow limitation, primarily caused by chronic inflammation in response to harmful inhalants such as tobacco smoke, air pollution, and occupational dust. The disease encompasses chronic bronchitis and emphysema, leading to symptoms like dyspnea, chronic cough, and sputum production, which worsen over time and significantly impair quality of life (Akdeniz and Özkan 2024). Globally, COPD affects approximately 480 million individuals, with a prevalence of 10.6% among those aged 25 or older (Boers et al. 2023). As the third leading cause of death worldwide, COPD accounts for 3.23 million annual deaths, with projections indicating it may rise further due to aging populations and persistent environmental risk factors (Safiri et al. 2022). The economic and societal burden of these conditions highlights the urgency of identifying modifiable risk factors and implementing preventive strategies to improve quality of life and reduce healthcare expenditures.

Depression, a highly prevalent mental health disorder, has emerged as a significant contributor to gastrointestinal pathophysiology through complex bidirectional mechanisms (Sonali et al. 2022). Accumulating evidence suggests that chronic depressive states may potentiate digestive disorders via multiple pathways, including sustained gastrointestinal inflammation, gut microbiota dysbiosis, and compromised intestinal barrier integrity, thereby predisposing to or exacerbating conditions such as irritable bowel syndrome and peptic ulcer disease (Leigh et al. 2023; Clapp et al. 2017). The underlying pathophysiology appears to involve maladaptive neuroendocrine responses, particularly hypothalamic‐pituitary‐adrenal axis hyperactivity coupled with elevated proinflammatory cytokine release (Rusch et al. 2023). Notably, this psychophysiological interplay extends to respiratory medicine, where depression demonstrates particularly high prevalence among patients with COPD and asthma. Recent epidemiological and mechanistic studies have revealed a robust bidirectional association between depressive disorders and respiratory conditions, suggesting shared pathophysiological pathways (Ran et al. 2023).

Arterial stiffness, a key indicator of vascular aging and cardiovascular risk, reflects the loss of arterial elasticity due to structural changes in the vessel wall. Increased arterial stiffness is strongly associated with hypertension, atherosclerosis, and elevated risks of myocardial infarction and stroke (Palombo and Kozakova 2016; Boutouyrie et al. 2021). It is also recognized as an independent predictor of cardiovascular mortality and morbidity (Xuereb et al. 2023). Emerging evidence highlights a significant association between arterial stiffness and respiratory diseases. Studies demonstrate that reduced lung function correlates with higher arterial stiffness, possibly due to systemic inflammation, oxidative stress, and shared risk factors such as smoking (Zanoli and Vancheri 2022; Jankowich et al. 2010). In hypertensive patients, increased arterial stiffness (measured by CAVI) is independently linked to impaired pulmonary function, including a lower FEV1/FVC ratio and elevated “pulmonary age,” a marker of accelerated lung aging (Masugata et al. 2012). Furthermore, preserved ratio impaired spirometry (PRISm), a condition with normal FEV1/FVC but reduced FEV1, is associated with greater arterial stiffness, suggesting that early lung function decline may contribute to vascular dysfunction (Kaufmann et al. 2024). However, the temporal relationship between arterial stiffness and COPD remains unexplored, with few longitudinal studies addressing this interplay in aging Asian populations.

This study employs data from the China Health and Retirement Longitudinal Study (CHARLS) to examine the associations between depression, ePWV, and their combined impact on the incidence of COPDs in older adults. By clarifying relationships between vascular health, depressive symptoms, and COPD risk, this study may help generate hypotheses that inform future research on integrated care approaches; however, direct testing of combined mental‐pulmonary interventions was beyond the scope of the present observational analysis.

## 2 Materials and Methods

### 2.1 Study Design and Participants

This study constitutes a secondary analysis of data obtained from the China Health and Retirement Longitudinal Study (CHARLS), a nationally representative prospective cohort encompassing Chinese adults aged ≥45 years (http://charls.pku.edu.cn/). The CHARLS employed a multistage probability sampling strategy, recruiting participants from 150 counties/districts and 450 villages across 28 Chinese provinces, with longitudinal follow‐up conducted between 2011 and 2020 (Zhao et al. 2014).

**FIGURE 1:**
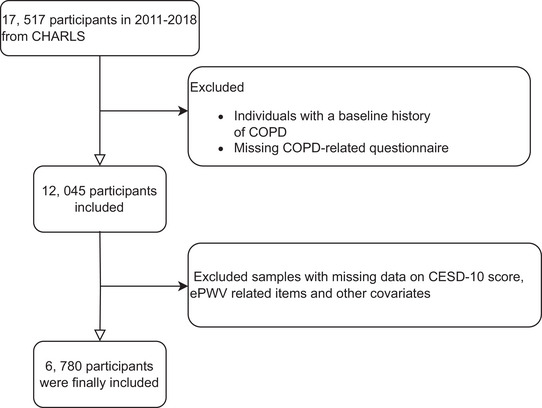
Flowchart of participant screening.

Our analysis incorporated data from CHARLS waves 1–4 (2011–2018), excluding wave 5 (2020) to mitigate potential COVID‐19‐related confounding. From the initial wave 1 cohort (*N* = 17,517), we excluded (1) participants with pre‐existing COPD or indeterminate COPD status at baseline and (2) cases with missing data on COPD status, estimated pulse wave velocity (ePWV), the 10‐item Center for Epidemiologic Studies Depression Scale (CESD‐10) scores, or other critical covariates. Additional exclusions addressed missing data on sociodemographic factors (educational attainment, residential status), behavioral characteristics (smoking status, alcohol consumption), and clinical parameters (hemoglobin, uric acid levels, diabetes mellitus, and heart disease status). This rigorous selection process, detailed in Figure 1, optimized data quality and analytical robustness.

### 2.2 Assessment of Depression and Arterial Stiffness

Depressive symptoms were measured in the 2011 wave using the CES‐D‐10, a validated and widely implemented self‐report instrument for depression screening in population‐based studies (Chen and Mui 2014). Each item is rated on a 4‐point Likert scale ranging from 0 (“rarely or none of the time”) to 3 (“most or all of the time”), with total scores ranging from 0 to 30. Consistent with established clinical thresholds, participants scoring ≥10 were classified as having clinically significant depressive symptoms (Pei et al. 2024; Zhang et al. 2024). Arterial stiffness was assessed using ePWV, a non‐invasive and practical surrogate marker derived from age and mean arterial pressure (MAP) (Jackson et al. 1992; Greve et al. 2016). The ePWV calculation has been validated against directly measured carotid‐femoral pulse wave velocity (cfPWV) and provides an accessible means to evaluate arterial health in large populations (Tan et al. 2023). In this study, ePWV was computed using the following formula: ePWV  =  9.587 − 0.402 × age  +  4.560  ×  10^− 3^ × age^2^ − 2.621  ×  10^− 5^ × age^2^ × mean blood pressure (MBP)  +  3.176 × 10^− 3^ × age × MBP  −  1.832  ×  10^− 2^ × MBP (Liu et al. 2025). In this formula, the mean blood pressure (MBP) was calculated as diastolic blood pressure (DBP)  +  0.4 × [systolic blood pressure (SBP) − DBP] (Liu et al. 2025).

### 2.3 Assessment of COPD and Their Follow‐up Time

COPD status was evaluated through participant interviews, including questions such as, “Have you been diagnosed with COPD (except for tumor or cancer) by a doctor?” The onset of COPD was recorded as the time of the initial diagnosis.

The incidence of COPD was assessed under different scenarios. For participants who did not report COPD at their most recent follow‐up, the event time was calculated as the interval between the last survey year and the baseline year. For those who developed COPD, the timing was determined based on the difference between the earliest reported onset year and the baseline year (Liu et al. 2025).

### 2.4 Covariate

This study accounted for key demographic, clinical, and behavioral covariates based on established criteria. Demographic factors included age, sex, waist circumference, residence (urban/rural), and education level (<high school/high school/≥college). Clinical biomarkers (uric acid, creatinine, hemoglobin, lipids, and glucose) were measured using standardized laboratory protocols (Huang et al. 2024). Chronic conditions were defined through physician diagnosis and/or biochemical thresholds: diabetes required either self‐reported diagnosis or elevated glucose/HbA1c (fasting glucose ≥ 125 mg/dL or HbA1c ≥ 6.5%) (Bai et al. 2024); cardiovascular diseases encompassed myocardial infarction, coronary heart disease, angina, heart failure, and related conditions; dyslipidemia was defined by abnormal lipid levels (triglycerides ≥ 2.3 mmol/L, total cholesterol ≥ 6.2 mmol/L, LDL‐C ≥ 4.1 mmol/L, or HDL‐C < 1.0 mmol/L); and hypertension was based on physician diagnosis or measured blood pressure ≥ 140/90 mmHg (Zhang et al. 2025, Jiang et al. 2024). Behavioral factors included smoking status (never/former/current) and alcohol consumption (never/ever). These comprehensive adjustments ensured robust control for potential confounders in all analyses (Yan et al. 2024, Chen et al. 2024). The selection of covariates was guided by prior epidemiological evidence to reduce confounding in association analyses. This study was not designed to establish causal effects, and no causal modeling framework was assumed.

### 2.5 Statistical Analysis

Descriptive statistics were computed for all study variables, with continuous variables presented as mean ± standard deviation (SD) for normally distributed data or median with interquartile range for non‐normally distributed variables. Categorical variables were summarized using frequency counts and percentages. Comparative analyses of baseline characteristics employed appropriate statistical tests: chi‐square tests for categorical variables, analysis of variance (ANOVA) for normally distributed continuous variables, and Kruskal–Wallis rank‐sum tests for nonparametric comparisons (Zhai et al. 2024).

The observation period for each participant extended from the baseline survey (2011–2012) until either COPD diagnosis or the conclusion of follow‐up. Cox proportional hazards regression models were implemented to quantify associations between depression, frailty, and COPD outcomes, expressed as hazard ratios (HRs) with 95% confidence intervals (CIs). Three hierarchical models were specified: an unadjusted model (Model 0), a partially adjusted model controlling for ePWV, hemoglobin, creatinine, uric acid, and sex (Model 1), and a fully adjusted model incorporating additional covariates including smoking status, hypertension, heart disease, and body mass index (Model 2). Potential nonlinear relationships were examined using 3‐knot restricted cubic spline regression analyses.

Participants were stratified into four exposure groups based on combined ePWV and depression status to evaluate their synergistic effects on COPD risk. The low‐ePWV, non‐depressed group served as the reference category for hazard ratio calculations. Survival probabilities were estimated using Kaplan‐Meier methodology, with corresponding curves presented in the results. Multivariable Cox regression analyses further elucidated independent risk factors for COPD incidence.

Mediation analyses were conducted to decompose the total effect of depression on COPD into direct and indirect pathways mediated by ePWV, with reciprocal analyses examining depression as a mediator in the frailty‐COPD relationship. All statistical procedures were executed in R software (version 4.2.1), utilizing specialized packages for mediation analysis (“mediation”) and survival modeling (“survival”). Statistical significance was determined using a conventional two‐tailed alpha threshold of 0.05 (Huo et al. 2024).

## 3 Results

### 3.1 Study Participants and Baseline Characteristics

The final cohort comprised 6,780 adults, including 718 participants diagnosed with new‐onset COPDs (Table 1). Compared to the non‐COPD group, individuals with COPD exhibited a higher ePWV and elevated CESD‐10 scores. Additionally, they showed lower baseline BMI, older age, and higher creatinine, uric acid, and hemoglobin levels, alongside a greater proportion of comorbid heart disease and hypertension. No significant differences were observed in educational attainment, residential distribution, or fasting glucose levels between the two groups (all *P* > 0.05).

**TABLE 1:** Baseline characters of participant.

	Overall (*n* = 6780)	No COPD (*n* = 6062)	COPD (*n* = 718)	*p*‐value
Age (years)	57.99 ± 8.96	57.77 ± 8.92	59.89 ± 9.09	< 0.0001
Sex (Male %)	2967(43.76)	2592(42.76)	375(52.23)	< 0.0001
BMI (kg/m^2^)	23.68 ± 3.78	23.72 ± 3.73	23.28 ± 4.17	< 0.01
Hemoglobin (g/dL)	14.34 ± 2.22	14.32 ± 2.21	14.52 ± 2.29	0.03
Glucose (mg/dL)	109.54 ± 33.90	109.60 ± 34.08	108.97 ± 32.39	0.62
Creatinine (mg/dL)	0.77 ± 0.19	0.77 ± 0.18	0.80 ± 0.22	< 0.001
Uric acid (mg/dL)	4.38 ± 1.22	4.37 ± 1.22	4.52 ± 1.26	< 0.01
Waist (cm)	84.38 ± 12.64	84.39 ± 12.65	84.35 ± 12.53	0.94
ePWV	9.33 ± 1.83	9.29 ± 1.81	9.71 ± 1.93	< 0.0001
TC (mg/dL)	193.36 ± 38.21	193.43 ± 38.20	192.79 ± 38.25	0.67
HDL‐C (mg/dL)	50.98 ± 15.02	50.87 ± 14.82	51.91 ± 16.58	0.11
LDL‐C (mg/dL)	116.59 ± 34.94	116.83 ± 34.86	114.53 ± 35.53	0.10
TG (mg/dL)	132.09 ± 95.91	132.01 ± 96.33	132.84 ± 92.32	0.82
Education (%)				0.05
Less than High School	6106 (90.06)	5451 (89.92)	655 (91.23)	
College	81 (1.19)	79 (1.30)	2 (0.28)	
High School	593 (8.75)	532 (8.78)	61 (8.50)	
Residence				0.12
Rural	4534 (66.87)	4035 (66.56)	499 (69.50)	
Urban	2246 (33.13)	2027 (33.44)	219 (30.50)	
Smoke status (%)				< 0.0001
Former, now quit	501 (7.39)	419 (6.91)	82 (11.42)	
Never	4296 (63.36)	3908 (64.47)	388 (54.04)	
Current	1983 (29.25)	1735 (28.62)	248 (34.54)	
Alcohol drink (%)	2217 (32.70)	1973 (32.55)	244 (33.98)	0.46
Dyslipidemia (%)				0.76
No	4030 (59.44)	3599 (59.37)	431 (60.03)	
Yes	2750 (40.56)	2463 (40.63)	287 (39.97)	
Diabetes Mellitus (%)	932 (13.75)	834 (13.76)	98 (13.65)	0.98
Heart disease (%)	657 (9.69)	561 (9.25)	96 (13.37)	< 0.001
Hypertension (%)	2653 (39.13)	2343 (38.65)	310 (43.18)	0.02
CESD‐10 score	8.26 ± 6.27	8.08 ± 6.21	9.77 ± 6.52	< 0.0001
Depression (%)	2483 (36.62)	2145 (35.38)	338 (47.08)	< 0.0001
Follow up time (years)	6.72 ± 1.10	7.00 ± 0.00	4.37 ± 2.29	< 0.0001

**Abbreviations**: BMI, body mass index; CESD‐10, the 10‐item Center for Epidemiologic Studies Depression Scale; COPD, chronic obstructive pulmonary disease; ePWV, estimated pulse wave velocity; HDL, high‐density lipoprotein; LDL, low‐density lipoprotein; TC, total cholesterol; TG, triglycerides.

### 3.2 Correlation between Depression, ePWV, and New‐Onset COPD

**FIGURE 2:**
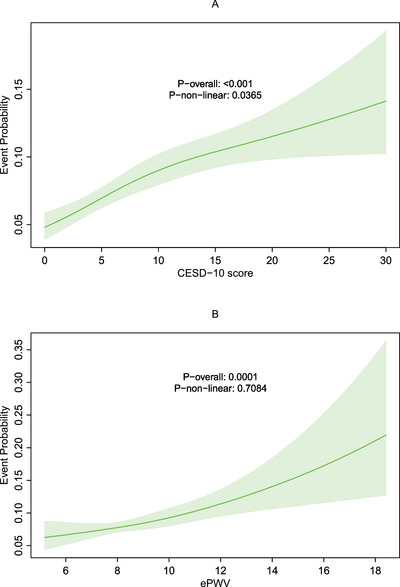
Restricted cubic spline (RCS) for the association between **(A)** CESD‐10 score and **(B)** ePWV with the risks of new‐onset COPD.

RCS analyses revealed distinct patterns of association between risk factors and COPD incidence. As shown in Figures 2A and 2B, CESD‐10 scores exhibited both a significant linear association (P overall < 0.001) and a non‐linear relationship (P non‐linear = 0.0365) with COPD risk. In contrast, while ePWV demonstrated a strong linear association with COPD risk (P overall < 0.001), our analyses did not detect significant non‐linearity in this relationship (P non‐linear > 0.05).

### 3.3 Associations of Depression, ePWV, and Their Combined Effect on COPD

**FIGURE 3:**
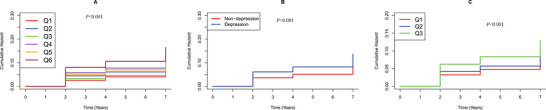
Kaplan Meier plot of COPD by CESD‐10 score and ePWV subgroups. **(A)** Categorized by joint variable of CESD‐10 score and ePWV; **(B)** Categorized by CESD‐10 score; and **(C)** Categorized by ePWV.

The multivariable‐adjusted Cox regression models and Kaplan‐Meier curves demonstrated significant associations between arterial stiffness, depressive symptoms, and COPD risk across different analytical approaches (Figure 3). When modeled as a continuous variable, each unit increase in ePWV was independently associated with an 11% higher risk of incident COPD (adjusted HR = 1.11, 95% CI 1.06–1.16, *P* < 0.0001). In categorical analyses, participants in the highest ePWV tertile (Q3) showed a 45% greater COPD risk compared to the lowest tertile (Q1) (adjusted HR = 1.45, 95% CI 1.16–1.80, *P* < 0.001). Similarly, continuous analysis of depressive symptoms revealed that each point increase in CESD‐10 score conferred a 4% higher COPD risk (adjusted HR = 1.04, 95% CI 1.03–1.06, *P* < 0.0001). When examining depression categorically, participants meeting depression criteria had 63% greater COPD incidence than non‐depressed individuals (adjusted HR = 1.63, 95% CI 1.40–1.90, *P* < 0.0001). Most notably, joint exposure analysis demonstrated a dose‐response relationship, with participants exhibiting both high ePWV and depression (Q6) experiencing the greatest risk elevation (adjusted HR = 2.17, 95% CI 1.65–2.68, *P* < 0.0001) compared to the reference group with low ePWV and no depression (Q1) (Table 2).

**TABLE 2:** Risk classification of new‐onset COPD based on ePWV and depression by Multiple Cox Regression analysis.

ePWV	Model 0 1.12 (1.08, 1.16)***	Model 1^a^ 1.11 (1.07, 1.15)***	Model 2^b^ 1.11 (1.06, 1.16)***
Low‐ePWV	Ref	Ref	Ref
Medium‐ePWV	1.24 (1.03, 1.51)*	1.19 (0.99, 1.45)	1.19 (0.97, 1.45)
High‐ePWV	1.6 (1.33, 1.92)***	1.51 (1.25, 1.81) ***	1.45 (1.16, 1.80)***
CESD‐10 score	1.04 (1.03, 1.05)***	1.05 (1.03, 1.06) ***	1.04 (1.03, 1.05)***
Non‐depression	Ref	Ref	Ref
Depression	1.58 (1.37, 1.83)***	1.72 (1.49, 2.00)***	1.63 (1.40, 1.90)***
Joint variable			
Q1	Ref	Ref	Ref
Q2	1.35 (1.01, 1.81)*	2.33 (1.82, 2.99)*	1.39 (1.04, 1.86)*
Q3	1.11 (0.86, 1.43)	2.33 (1.82, 2.99)	1.04 (0.80, 1.36)
Q4	1.92 (1.48, 2.50)***	2.33 (1.82, 2.99)***	1.9 (1.45, 2.48)***
Q5	1.47 (1.15, 1.88)**	2.33 (1.82, 2.99)*	1.33 (1.01, 1.74)*
Q6	2.31 (1.81, 2.96)***	2.33 (1.82, 2.99)***	2.17 (1.65, 2.86)***

^a^ Model 1 adjusted for sex, hemoglobin, uric acid, creatinine.

^b^ Model 2 adjusted for sex, hemoglobin, uric acid, creatinine, smoke, heart disease, hypertension, and BMI.

***p < 0.001, **p < 0.01, and *p < 0.05.

Q1: low‐ePWV + non‐depression.

Q2: low‐ePWV + depression.

Q3: medium‐ePWV + non‐depression.

Q4: medium‐ePWV + depression.

Q5: high‐ePWV + non‐depression.

Q6: high‐ePWV + depression.

### 3.4 Mediation Analyses of Frailty and Depression in COPD

**FIGURE 4:**
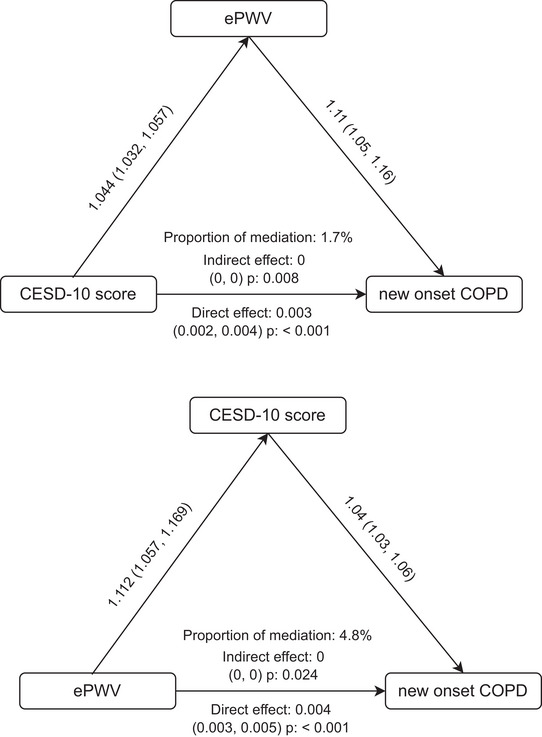
Mediation analyses of CESD‐10 score and ePWV on new‐onset COPD.

Mediation analysis (Figure 4) indicated bidirectional effects. ePWV mediated 1.7% of the association between depression and COPDs (indirect effect *P* = 0.008), while depression mediated 4.8% of the ePWV‐COPD link (indirect effect *P* = 0.024), underscoring their interconnected roles in disease pathogenesis.

## 4 Discussion

Given the observational design, these associations cannot establish causal direction. Our study suggests that both ePWV and depression independently contribute to the risk of COPD, with potentially compounding effects when these factors co‐occur. Emerging evidence demonstrates a complex bidirectional relationship between depression and respiratory diseases (Soriano et al. 2017), with depression both contributing to and exacerbating conditions such as COPD, asthma, and obstructive sleep apnea (OSA). This association appears to be mediated through multiple interconnected biological and behavioral pathways. At the biological level, chronic systemic inflammation serves as a key mechanistic link (Miller and Raison 2016), as both depression and respiratory diseases are characterized by elevated pro‐inflammatory cytokines, including interleukin‐6 (IL‐6), tumor necrosis factor‐alpha (TNF‐α), and C‐reactive protein (CRP). These inflammatory markers not only contribute to neurochemical changes in the brain that underlie depression but also exacerbate airway inflammation and lung tissue damage (Karadag et al. 2008, Felger and Lotrich 2013, Solmi et al. 2021). Hypoxia represents another critical pathway, as chronic oxygen deprivation in respiratory diseases may lead to neuronal damage in mood‐regulating brain regions such as the hippocampus and prefrontal cortex (Barnes 2017, Zhao et al. 2017). Furthermore, dysregulation of the hypothalamic‐pituitary‐adrenal (HPA) axis in depression results in excessive cortisol secretion, which can impair immune function and worsen respiratory symptoms (Miller and Raison 2016). From a behavioral perspective, depression frequently leads to poor adherence to respiratory medications and pulmonary rehabilitation, reduced physical activity, and increased smoking rates—all of which negatively impact disease progression (Ran et al. 2023, Rahi et al. 2023). Notably, the relationship appears to be bidirectional (Wickwire et al. 2024), as respiratory symptoms like dyspnea and sleep disturbances can trigger or worsen depressive symptoms, creating a vicious cycle of disease exacerbation. Shared genetic vulnerabilities may also contribute to this comorbidity, with certain polymorphisms in genes related to serotonin transport and inflammatory regulation being associated with both conditions (Reith et al. 2022, Cabana‐Domínguez et al. 2022). The clinical implications of this relationship are substantial, as comorbid depression in respiratory patients is associated with more severe symptoms, increased exacerbation frequency, longer hospital stays, higher healthcare utilization, and greater mortality risk (Ran et al. 2023, Yohannes and Alexopoulos 2014). These findings highlight the need for integrated care approaches that address both mental health and respiratory management, with particular attention to early screening for depression in respiratory patients and consideration of anti‐inflammatory treatment strategies that may benefit both conditions.

Increased arterial stiffness, as measured by ePWV, is also closely associated with respiratory diseases, particularly COPD and obstructive sleep apnea syndrome (OSAS) (Galerneau et al. 2020, Clímaco et al. 2023). The pathological link may involve several interconnected mechanisms. First, systemic inflammation, a hallmark of chronic respiratory diseases, promotes endothelial dysfunction and vascular remodeling, leading to increased arterial stiffness (Karadag et al. 2008, Pacinella et al. 2022, Mozos et al. 2017). Conversely, elevated arterial stiffness may impair pulmonary circulation by increasing pulsatile pressure transmission to the microvasculature, potentially exacerbating pulmonary hypertension and right ventricular dysfunction (Sommer et al. 2008). Hypoxemia in chronic respiratory diseases may further accelerate vascular stiffening through oxidative stress‐mediated vascular damage and sympathetic nervous system activation (Sommer et al. 2008, Ragnoli et al. 2025). The shared risk factors, particularly smoking, contribute to both pulmonary and vascular pathology through chronic inflammation and oxidative stress pathways (Barnes 2017). Moreover, arterial stiffness may worsen respiratory symptoms by reducing cardiovascular reserve during physical activity, thereby limiting exercise capacity in patients with pre‐existing lung disease (Hermann et al. 2025). These findings highlight the importance of vascular assessment in respiratory patients and suggest that interventions targeting arterial stiffness may improve both cardiovascular and pulmonary outcomes.

Currently, there is limited direct evidence examining the joint effects of arterial stiffness (as measured by estimated pulse wave velocity, ePWV, and depression on respiratory diseases or all‐cause mortality. However, separate studies suggest that both factors independently contribute to adverse health outcomes, and their combined influence may exacerbate disease progression. Mechanistically, systemic inflammation and endothelial dysfunction may serve as common pathways, where chronic inflammation in depression could further accelerate vascular stiffness, while arterial stiffness may impair pulmonary circulation and worsen respiratory function (Karnati et al. 2021). Future research should investigate whether interventions targeting both vascular health and mental well‐being could improve outcomes in this high‐risk population.

This study highlights the interplay between vascular stiffness (ePWV), depressive symptoms, and COPD risk. The independent and combined associations observed in this cohort indicate that concurrent assessment of vascular health and mental well‐being may improve risk stratification in populations at elevated risk of COPD, particularly older adults with cardiometabolic comorbidities. Although individuals presenting with both higher ePWV and depressive symptoms exhibited a graded increase in COPD risk (HR = 2.17), this pattern should be interpreted as an epidemiological observation rather than evidence supporting specific clinical interventions. The mediation analyses were performed to characterize statistical interrelationships between exposures rather than to infer causal pathways. Although the indirect effects were statistically significant, the proportions mediated were small (1.7% and 4.8%), indicating limited clinical or public health relevance. These findings do not support a causal mediation interpretation but instead suggest that arterial stiffness and depressive symptoms share only modest overlapping pathways in relation to new‐onset self‐reported chronic obstructive pulmonary disease. The majority of the observed associations are therefore likely attributable to other unmeasured or parallel mechanisms.

This study possesses several notable strengths. First, leveraging data from the CHARLS cohort enabled the inclusion of a large, nationally representative sample with longitudinal follow‐up, thereby enhancing the robustness of our findings regarding the associations between arterial stiffness, depression, and COPD risk. Second, the application of mediation analysis provided novel insights into the bidirectional relationship between ePWV and depressive symptoms in the pathogenesis of COPD. Several limitations should be acknowledged. First, given the observational design, this study was intended to assess exposure–outcome associations for risk stratification rather than to establish causal effects or estimate disease‐specific incidence. Although mortality may act as a competing event for clinically diagnosed chronic obstructive pulmonary disease, the outcome of interest was self‐reported new‐onset COPD rather than survival or adjudicated incidence; therefore, competing risk modeling was not the primary focus of the present analysis. Second, both exposure and outcome measurements were subject to potential misclassification. COPD status was based on self‐reported physician diagnosis, which is susceptible to recall and reporting bias. Arterial stiffness was assessed using ePWV rather than directly measured carotid–femoral pulse wave velocity, and depressive symptoms were evaluated using the CESD‐10 screening instrument rather than clinical psychiatric assessment. These factors may have introduced non‐differential misclassification and biased the observed associations toward the null. Third, although a comprehensive set of demographic, behavioral, and clinical covariates was adjusted for, residual confounding cannot be excluded, particularly from unmeasured factors such as air pollution exposure, medication use, dietary patterns, and underlying cardiac or pulmonary function. Finally, the study population was derived from the CHARLS cohort, which primarily includes middle‐aged and older Chinese adults. Caution is therefore warranted when generalizing these findings to younger populations or other ethnic and geographic groups. Future studies incorporating objective clinical measurements, competing risk frameworks where appropriate, and more diverse populations are needed to validate and extend these findings.

Further investigation is warranted to elucidate the underlying biological mechanisms connecting arterial stiffness, depression, and COPD risk. Explorations into potential mediators, such as systemic inflammation and neuroendocrine dysregulation, may yield deeper mechanistic understanding. Additionally, clinical trials evaluating the efficacy of integrated interventions—encompassing nutritional optimization, structured physical activity, psychological therapies, and pulmonary rehabilitation—could inform evidence‐based strategies to mitigate COPD progression in older adults. Targeting these modifiable risk factors may ultimately facilitate the development of precision medicine approaches in the management of respiratory diseases.

## 5 Conclusion

Our findings indicate independent associations of ePWV and depressive symptoms with COPD incidence, with small indirect effects in mediation analyses. These results are exploratory and hypothesis‐generating rather than confirmatory. Future research should validate these associations using measured PWV, clinical psychiatric assessments, and experimental designs.

## Author Contributions

Q. Q. S. and X. Y. Z. conceived and designed the study, acquired the data, and drafted the manuscript. T. L. analyzed the data. T. S. L. and Y. J. X. contributed to the interpretation of the results and critical revision of the manuscript for important intellectual content. T. L. and H. X. W. developed the software and provided technical support. T. L. and H. X. W. had the primary responsibility for final content. All authors have read and approved the final manuscript.

## Funding

The authors have nothing to report.

Ethics Statement

The CHARLS study adhered to the ethical principles outlined in the Declaration of Helsinki and received approval from the Institutional Review Board of Peking University (IRB00001052‐11015). The research involving human participants was approved by the Ethics Committee of Peking University. We also received approval from the Institutional Review Board of Zhuji Affiliated Hospital of Wenzhou Medical University (2024 [1021]).

## Consent

Written informed consent was obtained from all patients/participants prior to their involvement in the study.

## Conflicts of Interest

The authors declare no conflicts of interest.

## Data Availability

The datasets used and/or analyzed in this research are publicly accessible or can be obtained from the corresponding author upon reasonable request at http://charls.pku.edu.cn/en.
